# Integrated Pharmacology Reveals the Molecular Mechanism of Gegen Qinlian Decoction Against Lipopolysaccharide-induced Acute Lung Injury

**DOI:** 10.3389/fphar.2022.854544

**Published:** 2022-05-13

**Authors:** Wei Li, Zihe Ding, Ying Chen, Yi Wang, Mingming Peng, Chuanqiu Li, Han Zhang, Renxing Zhong, Tianyi Xia, Luyang Zhong, Mantong Zhao, Mengru Yang, Yimin Yue, Lanyuan Liang, Xia Cao, Zunpeng Shu

**Affiliations:** ^1^ The College of Traditional Chinese Medicine, Guangdong Pharmaceutical University, Guangzhou, China; ^2^ School of Pharmacy, Jiamusi University, Jiamusi, China

**Keywords:** gegen qinlian decoction, acute lung injury, immunity, intestinal microbiota, multi-omics

## Abstract

ALI is a severe inflammatory disease of the lungs. In previous studies, we found that GQD was effective against ALI, but specific molecular mechanism is still unclear. Therefore, this study was to examine effect of GQD on LPS-induced ALI rats and underlying mechanisms using multi-omics and molecular methods. The results showed that GQD significantly improved lung tissue damage, reduced pulmonary edema, inhibited MPO activity, and improved respiratory function in ALI rat. Additionally, GQD significantly reduced the levels of TNF-α, IL-1β, and IL-6 in serum and BALF. Furthermore, metabolomic analysis showed that GQD reduced pulmonary inflammation by improving metabolic remodeling. Moreover, transcriptomic analysis showed that GQD inhibited the activation of complement pathway and regulated Th17 and Treg cells balance. Additionally, GQD inhibited the expression of C3, C5a, and IL-17, and promoted the expression of TGF-β and CYP1A1 at the mRNA and protein levels. Gut microbial assay showed that GQD treatment increased the relative abundance of Firmicutes and their genera in intestinal microbiota, and increased short-chain fatty acids concentration. Overall, GQD treated ALI by improving metabolic remodeling, affecting immune-related pathways and regulating intestinal microbiota. This study provides a solid scientific basis for promoting the clinical use of GQD in treating ALI.

## 1 Introduction

Acute lung injury (ALI) is a critical respiratory disease characterized by pulmonary inflammation and structural destruction of lung tissue (Wang, 2019). Studies have shown that immune cell dysfunction and imbalance of immune inflammatory factors play significant roles in the occurrence and development of ALI (Kim et al., 2017). The complement system is an important component of the non-specific immune system, and its abnormal activation can cause serious pathological damage to body tissues (Ming et al., 2016). As a key molecule of complement immunity, C5a is an important mediator and chemotactic factor of inflammatory response, which can cause chemotaxis, aggregation, and activation of immune cells in the lung, initiating and amplifying inflammatory response, thus damaging lung tissue (Wang et al., 2015). Additionally, a balance between Th17 and Treg cells is crucial for maintenance of immune homeostasis and an imbalance is associated with a variety of inflammatory respiratory diseases (Sun et al., 2019; Li et al., 2020; Sadeghi et al., 2020). Th17 cells secrete inflammatory factors, such as IL-17 and IL-23, which mediate inflammatory response (Gai-Jun et al., 2021a). In contrast, Treg cells mainly inhibit the proliferation and differentiation of T cells by secreting cytokines such as TGF-β, thus participating in the regulation of immune responses (Dan and Rudensky, 2010). Although these two immune pathways are involved in the progression on ALI, they also represent research targets for the development of anti-ALI drugs.

Intestinal microbial composition has been linked to immune response and inflammation, and play an essential role in the pathological immune response of intestines and lungs (Yun et al., 2020). Additionally, gut microflora indirectly regulates systemic immunity by regulating T cell population and short-chain fatty acids concentration (Blander et al., 2017). For example, the genus of Firmicutes participate in the fermentation of dietary components and the production of short-chain fatty acids (SCFAs), which can help maintain the intestinal mucosal barrier (Markowiak-Kopeć and Śliżewska, 2020). Some studies have confirmed that the lungs and intestines influence each other under physiological and pathological conditions, resulting in the concept of the gut-lung axis (He et al., 2017; Dang and Marsland, 2019). An imbalance in intestinal flora can cause changes in the immune system through the interaction of the gut-lung axis, eventually leading to acute and chronic lung diseases (Yimin et al., 2017). Based on the gut-lung axis theory, recent studies on drug development have focused on treating lung diseases by improving intestinal microflora.

Traditional Chinese medicines (TCM) have been used in the treatment of diseases for a long history; however, the mechanisms of action are poorly understood. With the rapid development of high-throughput sequencing technology, transcriptomics, metabolomics, and 16s rDNA sequencing, research outcomes on complex disease systems has improved considerably (Moni and Liò, 2015). Multi-omics technology can be used to understand the internal relationship between tissue structure, function, biological macromolecules, and endogenous small molecules (Qiong et al., 2020). Multi-omics technology can be used to examine the complexity of molecules at several levels, which can be used to understand the relationship between human health and diseases, and to elucidate the molecular mechanism of TCM disease treatment of diseases (Gai-Jun et al., 2021b). Therefore, multi-omics technology provides a new means to explore the treatment mechanism of traditional Chinese medicine compound prescription.

Gegen Qinlian decoction (GQD) is a prescription of traditional Chinese medicine, that is often used in treating intestinal and pulmonary inflammatory diseases (Dayong, 2018; Shiheng, 2020). It is composed of *Puerariae lobatae radix*, *Scutellariae radix*, *Coptidis rhizoma*, and *Glycyrrhizae radix et rhizome.* Recent studies have found that GQD was effective against ALI (Jinfu et al., 2015). In the early stage, we also used the method of network pharmacology to predict that GQD could improve ALI mainly through cell survival pathway and immune regulation pathway, and we established a model of ALI mice induced by intraperitoneal injection of LPS. By using the method of transcriptome, we found that GQD mainly inhibited cell apoptosis through the PI3K/Akt survival pathway, thus alleviating the disease of lung injury in ALI mice (Ding et al., 2020). In this study, another method was used to induce ALI in rats by intratracheal injection of LPS. We used metabolomics, transcriptomics and 16s rDNA technology to explore different modeling methods, whether GQD could improve ALI through the same mechanism, so as to provide a solid scientific research basis for clinical use of GQD.

## 2 Materials and Methods

### 2.1 Reagents

Lipopolysaccharide (LPS, *Escherichia coli* 055: B5) was purchased from Sigma-Aldrich (St. Louis, MO, United States). TNF-α, IL-1β and IL-6 ELISA kits were purchased from Jiancheng Biological Engineering Research Institute (Nanjing, China). Specific primers (Supplementary Table S1) for TNF-α, IL-1β and IL-6 determination in rats were purchased from Invitrogen Inc. (Carlsbad, CA. United States). TRIzol reagent was purchased from Life Technologies Company. HiPure Stool DNA Kits were purchased from Guangzhou Meiji Biotechnology Co., Ltd., China. Specific primers for C3, C5aR1, IL-17A, TGF-β, and CYP1A1 determination in rats were purchased from Invitrogen Inc. (the primers are shown in Supplementary Table S2). C3 rabbit antibody, C5a rabbit antibody, and CYP1A1 rabbit antibody were purchased from Chengdu Zhengneng Biology Co., Ltd. TGF-β rabbit anti, IL-17 rabbit anti, HRP-labeled goat anti-rabbit secondary antibody, and histochemical kit DAB chromogenic agent were purchased from Wuhan Seville Biological Co., Ltd.

### 2.2 Drug Preparation

GQD was composed of *Puerariae lobatae radix*, *Scutellariae radix*, *Coptidis rhizoma*, and *Glycyrrhizae radix et rhizoma*. The four medicinal materials for GQD were purchased from Kangmei Pharmaceutical Co., Ltd. (Guangzhou, China), and identified by Professor Li Shuyuan from the Department of Traditional Chinese Medicine Resources, Guangdong Pharmaceutical University. The extract of GQD was prepared according to previous research conditions (Ding et al., 2020), using 250 g of *Puerariae lobatae radix*, 150 g of *Scutellariae radix*, 150 g of *Coptidis rhizoma*, and 100 g of *Glycyrrhizae radix et rhizoma*. Briefly, *Puerariae lobatae radix* was soaked in 2 L of cold water for 0.5 h, decocted alone for 0.5 h. The other herbs, including *Scutellariae radix*, *Coptidis rhizoma*, and *Glycyrrhizae radix et rhizome* were added and decocted together with *Puerariae lobatae radix* for 1 h, filtered with gauze, and cooled. The first decoction was thus obtained. The filter residue was boiled a second time with 1.5 L of water for 1 h and filtered to obtain the second decoction. Finally, the first and second decoctions were mixed and concentrated to a final extract. Distilled water was used to dissolve the liquid at a concentration of 25 mg/ml.

### 2.3 Establishment of LPS-Induced Acute Lung Injury Rat Model

One hundred and forty-four specific-pathogen free (SPF) male SD rats weighing 280 ± 20 g were purchased from the Experimental Animal Center of Guangzhou University of Chinese Medicine, Guangdong [animal license No. SCXK (Guangdong) 2018-0034]. All animal procedures were performed in accordance with the Guidelines for Care and Use of Laboratory Animals of Guangdong Pharmaceutical University (no. 20170142) and approved by the Animal Ethics Committee of Guangdong Pharmaceutical University. The animals were housed under standard conditions of alternating 12-h light and dark cycle at an ambient temperature of 22 ± 2°C, and relative humidity of 50 ± 5%. The animals had libitum access to food and water.

#### 2.3.1 Mechanism Exploration

To examine the mechanism of GQD against ALI, the rats were randomly assigned to six treatment groups according to their body mass (16 rats per group): control group (C), LPS model group (M, 7 mg/kg), GQD low (GL, 0.6 g/kg), medium (GM, 1.2 g/kg), high (GH, 2.4 g/kg) dose groups, and dexamethasone positive drug control group (DEX, 7 mg/kg). The rats were allowed to acclimate to the experimental environment, water, and feed for 1 week, and then administered GQD by intragastric injection according to the experimental doses. Rats in the control group and model group were administered the same volume of normal saline. After 3 days of intragastric administration, rats in the model and treatment groups received intratracheal injection of LPS (Liang et al., 2020), while those in the control group received the same dose of normal saline. The room temperature was maintained at approximate 25°C. After 48 h of modeling, the drug was administered every 24 h. Two hours after the last administration, blood samples were collected from the abdominal aorta of anesthetized rats. Blood serum was separated by centrifugation at 3000 rpm for 15 min and stored at −80°C for further analysis. Bronchoalveolar lavage fluid (BALF) and fecal samples were collected and lung tissues were harvested for further analysis.

#### 2.3.2 Mechanism Verification

The rats were randomly assigned into four treatment groups according to body weight, with 12 rats in each group: control group (C), LPS model group (M, 7 mg/kg), GQD treatment group (LPS + GH, 2.4 g/kg) and GQD single administration group (GH, 7 mg/kg). Modeling and sampling were performed according to the above-mentioned method.

### 2.4 Histopathological Observation of Lung

To evaluate histological changes in lung tissues, the tissues were washed three times with PBS, fixed in 4% paraformaldehyde, embedded in paraffin, cut into 4 μm, and stained with hematoxylin and eosin. Pathological changes in the lung tissues were observed under an Inverted biological microscope (DSZ2000X, Chongqing Aopu Optoelectronic Technology Co., Ltd.).

### 2.5 Determination of Lung Wet/Dry Weight Ratio

The wet/dry weight ratio was calculated to assess pulmonary edema. Briefly, the caudate lobe of the right lung weighed. Lung tissue was dried in an oven at 80°C for 48 h and then weighed to determine the baseline dry weight of lung.

### 2.6 Determination of Total Protein Concentration in BALF

BALF was collected by intratracheal administration of 1 ml PBS into the lung and pumped back and forth gently three times. BALF was centrifuged at 1500 rpm for 10 min at 4°C to pellet the cells. The total cells were resuspended in 100-μL PBS, and the protein concentration in the supernatant was determined according to the instructions of BCA protein determination kit.

### 2.7 Determination of Myeloperoxidase Activity in Lung Tissue

MPO activity in the lung tissues were determined using MPO detection kit according to the manufacturer’s instructions. Changes in optical density value were measured at 460 nm to calculate MPO activity, which was used to evaluate lung cell infiltration.

### 2.8 Respiratory Function Measurement

The external respiratory channel was established according to the animal experimental operation, and the airway was connected to the animal ventilator. The data were analyzed using a data analysis system with a 16-channel PowerLab data acquisition and analysis system, and the respiratory rate and respiratory cycle were observed.

### 2.9 Determination of TNF-α, IL-1β, and IL-6 Activity in the Serum and BALF

The serum and BALF supernatant frozen at-80°C were taken and the levels of TNF-α, IL-1β and IL-6 were quantified using the respective ELISA kit, according to the manufacturer’s instructions.

### 2.10 Determination of the Expression of Related Genes in Lung Tissue

The related genes in rat lung tissue were detected by real-time fluorescence quantitative PCR (RT-qPCR). The total RNA was isolated and purified with TRIzol reagent manual. The genomic DNA reaction was removed by Takara company kit, and the fluorescence quantitative PCR was detected according to PrimeScript RT kit and SYBR ®PremixExTaq II kit.

### 2.11 Immunohistochemical Analysis

The presence of C3, C5a, IL-17, TGF-β, and CYP1A1 were detected by immunohistochemical staining. Hydrated paraffin sections of lung tissue were incubated in a sealing solution (10% normal rabbit serum +5% skimmed milk powder +3% BSA +0.1% TritonX-100) for 10 min, and then the first antibody was added and incubated overnight at 4°C. After rinsing with PBS (pH 7.4), horseradish peroxidase (HRP)-goat anti-rabbit IgG antibody (1:200) was added and incubated at room temperature for 50 min. The sections were then incubated with diamino-benzidine on a chromogenic substrate, counterstained with hematoxylin, and visualized using inverted biological microscope (DSZ2000X, Chongqing Aopu Optoelectronic Technology Co., Ltd.). The extent of cell immunopositivity was assessed visually.

### 2.12 Metabolomics Analysis

#### 2.12.1 Preparation and Metabonomic Analysis of Lung Tissue Samples

Lung tissues from the control (C), LPS model (M, 7 mg/kg), GQD high-dose (GH, 2.4 g/kg) groups were used for metabolomics. Lung tissue samples (200 mg) were added to 1.5 ml ice-cold 50%/50% acetonitrile-water extraction system, homogenized using an electric homogenizer, and centrifuged at 12000 rpm for 20 min at 4°C. The upper layer was freeze-dried and then redissolved in sodium phosphate buffer (0.2 M Na_2_HPO_4_/NaH_2_PO_4_, containing 0.05% TSP, pH = 7.0) prepared with 600 μL of heavy water. The samples were incubated at room temperature for 20 min and then vortexed. The samples were centrifuged at 12,000 rpm for 10 min at 4°C to remove the precipitate. The supernatant (550 μl) was transferred into a 5-mm nuclear magnetic tube for inspection. All ^1^H-NMR spectra were recorded on a superconducting Fourier transform nuclear magnetic resonance (Bruker AVANCE III 500 MHz) at 25°C. The cumulative number of scans was 128, the number of sampling points was 64000, the spectral width was 20 kHz, and the sampling time was 2.54 s. Finally, the collected FID signals were multiplied by the corresponding exponential weight function (0.3 Hz linewidth), and then Fourier transformation was performed to obtain a one-dimensional ^1^H-NMR spectrum.

The peaks were calibrated by manual phase (apks) correction and baseline (abs) adjustment using Topspin3.0. The peaks of TSP (1H, δ 0.00) were used as chemical shift zeros, and the methyl peaks of lactic acid (CH3, δ 1.33) were calibrated. The data were saved for the follow-up analysis.

#### 2.12.2. Correlation Analysis of Metabolites in Lung Tissue

The quantitative metabolic data of each spectrum were normalized using the same proportional parameters in the multivariate analysis and then read into R software. The relationship between metabolites was determined by Pearson’s correlation analysis.

### 2.13 Transcriptome Sequencing and Analysis

#### 2.13.1 Isolation and Sequencing of RNA

Total RNA was isolated from lung tissues and purified according to the instructions of TRIzol reagent, according to the manufacturer’s instructions. RNA quantity and purity was assessed using nano-photometer. RNA integrity was assessed using the RNA Nano 6000 Assay Kit of the Bioanalyzer 2100 system (Agilent Technologies, CA, United States). A total of 1 μg RNA per sample was used as the input material for RNA sample preparation. Sequencing libraries were generated using NEBNext ®UltraTM RNA library Prep kit for Illumina ® (Nebraska, United States), according to the manufacturer’s instruction, and index codes were added to attribute sequences to each sample.

### 2.13.2 Data Analysis of Gene Expression

Differential expression analysis was performed using DESeq2R software (1.16.1). DESeq2 provides statistical procedures for determining the differential expression in digital gene expression data using a model based on negative binomial distribution. The resulting *p*-values were adjusted using Benjamini and Hochberg’s approach for controlling the false discovery rate. Genes with an adjusted *p*-value < 0.05 were assigned as differentially expressed. enrichment analysis of differentially expressed genes was performed using clusterProfiler (3.4.4) software, and pathways with corrected *p* < 0.05 were considered significantly enriched by the differentially expressed genes.

### 2.14 16s rDNA Gene Sequencing of Intestinal Flora

At the end of the treatments, fresh fecal samples were collected from the rats and stored at −80°C for 16S rDNA amplicon sequencing. Total DNA was extracted from fecal samples using fecal DNA extraction kit, according to the manufacturer’s instructions. The extracted DNA was stored at −20°C for further analysis Diluted genomic DNA was used as the template. Specific primers combined with adapter sequences and barcode sequences was used to amplify the specific region of the bacterial 16S rDNA gene, followed by two rounds of PCR. PCR products from the first step were purified using AMPure XP Beads and quantified using Qubit 3.0. PCR products from the second step were purified using AMPure XP Beads and quantified using ABI StepOnePlus Real-Time PCR System (Life Technologies, United States). The products were pooled together and sequenced on Illumina PE250 platform using Novaseq 6000 (San Diego, CA, United States).

### 2.15 Relationship Between 16s rDNA Sequence Data and Transcriptome Data

The relationship between RNA sequence data and transcriptome data was determined using Pearson’s correlation analysis. Pearson’s correlation can be used to measure the association between two variables, and the coefficient range from −1, to +1. The relationship between microorganism abundance and gene expression was examined by Pearson’s correlation using the COR. TEST function of R software, and *p*-values were calculated based on the Fisher-Z transform. Network analysis was performed using the R language igraph package (version 1.1.2), and the network diagram was further processed using Cytoscape software.

### 2.16 Determination of Short Chain Fatty Acids

The SCFAs content of the fecal samples was determined by Waters ACQUITY UPLC combined with AB SCIEX 5500 QQQ-MS in Guangzhou Gidio Biotechnology Co., Ltd. (Guangzhou, China). Briefly, SCFAs were extracted from feces using acetonitrile/aqueous solvent mixtures. Octanoic acid-1-13C1 was used as the internal standard for the precise quantification of individual SCFAs.

### 2.17 Statistical Analysis

All data are expressed as the mean ± standard deviation (SD). A Student’s t-test was performed, and *p* < 0.05 was considered statistically significant. Principal component analysis (PCA) and partial least-squares discriminant analysis (PLS-DA) were performed on Simcap 11.0 (Umetrics, Umea, Sweden). GraphPad Prism 8.0 software (GraphPad, CA, United States) was used for graphics.

## 3 Result

### 3.1 Gegen Qinlian Decoction has Protective Effect Against LPS-Induced Acute Lung Injury *in vivo*

**FIGURE 1:**
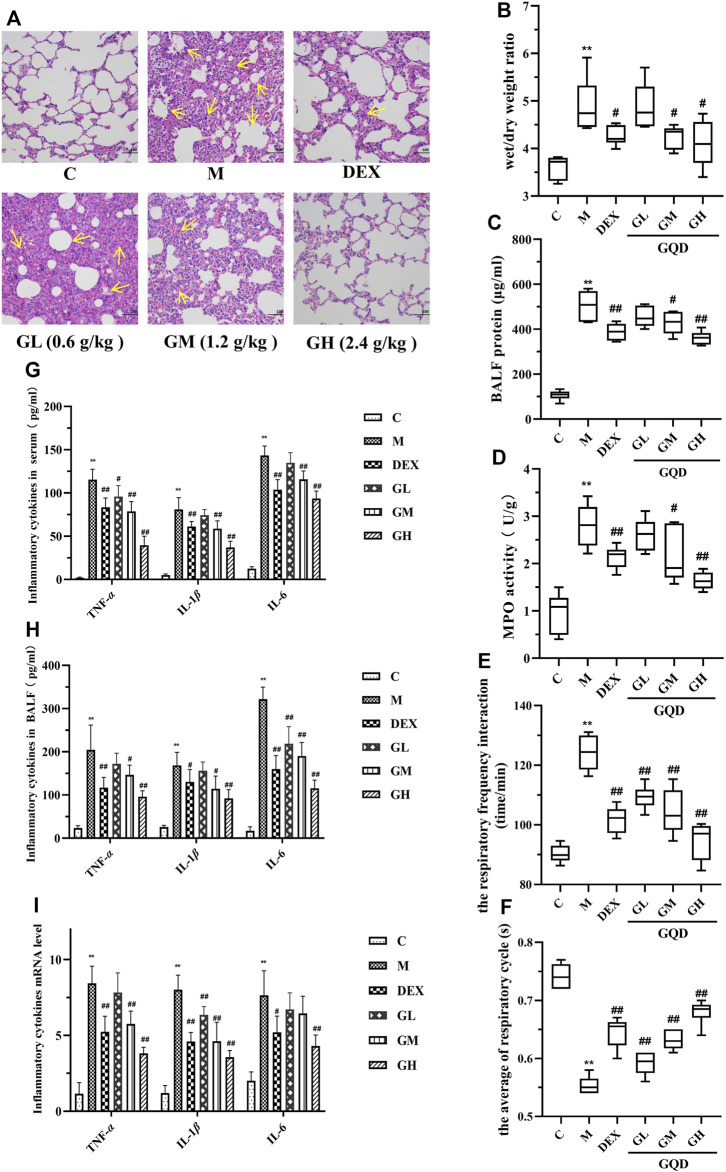
GQD has protective effect on ALI induced by LPS *in vivo* (*n* = 8). **(A)** Lung tissue sections were stained with hematoxylin and eosin **(H,E)** for histopathology analysis (magnification ×200). **(B)** Lung wet/dry weight ratio. **(C)** Total protein content in BALF. **(D)** MPO activity of lung tissue. **(E)** Changes in respiratory frequency. **(F)** Changes in respiratory cycle. **(G)** Cytokine activity in serum. **(H)** Cytokine activity in BALF. **(I)** Expression of cytokine mRNA in lung tissue. The values in the figure were expressed as mean ± standard deviation. Compared with the control group, **p < 0.01. Compare with model group, ^#^ *p <* 0.05, ^##^ *p <* 0.01.

Tracheal LPS-induced ALI is a recognized animal model, which can better simulate the clinical symptoms of ALI (Yi et al., 2021). In order to explore the intervention effect of GQD on ALI, histopathological examination of the lungs of GQD-treated LPS-induced ALI rats was performed. The results are shown in Figure 1A. The lung tissue of rats in the model group was characterized by alveolar interstitial edema, alveolar wall thickening, and high inflammatory cell infiltration into the alveoli. After intervention with GQD and positive drugs, the symptoms were significantly alleviated. The effect of high dose GQD was significantly better than that of other administration groups.

To further evaluate the protective effect of GQD against LPS-induce ALI, parameters related to pulmonary edema, microvascular permeability, and respiratory function were examined. The parameters included lung wet/dry weight ratio, protein content in BALF, myeloperoxidase (MPO) activity in lung tissue, respiratory frequency, and respiratory cycle. Compared with the control group, lung wet/dry ratio (Figure 1B), protein content in BALF (Figure 1C), MPO activity in lung tissue (Figure 1D), and respiratory function (Figures 1E,F) of rats in model group were negatively affected by ALI. However, GQD and positive drugs alleviated these LPS-induced pathological changes, with GQD treatment slightly more effective than that of positive drugs.

As local expression of pro-inflammatory TNF-α, IL-1β, and IL-6 plays key role in the ALI development (Lin and Yang, 2018), the activity and mRNA expression of these inflammatory factors were determined using ELISA kit and by RT-qPCR. Results showed that LPS-induced ALI significantly increased the activity and expression of TNF-α, IL-1β, and IL-6 in the serum and BALF of the experimental rats. However, GQD and positive drugs treatment reversed these effects, with high-dose GQD treatment having the best effect (Figures 1G–I). The above results indicate that a high dose of GQD could be effective in maintaining alveolus-vascular barrier integrity and respiratory function, and can effectively reduce excessive pulmonary inflammatory response in ALI rats.

### 3.2 Metabolomics of the Lungs Gegen Qinlian Decoction-Treated LPS-Induced Acute Lung Injury Rats

**FIGURE 2:**
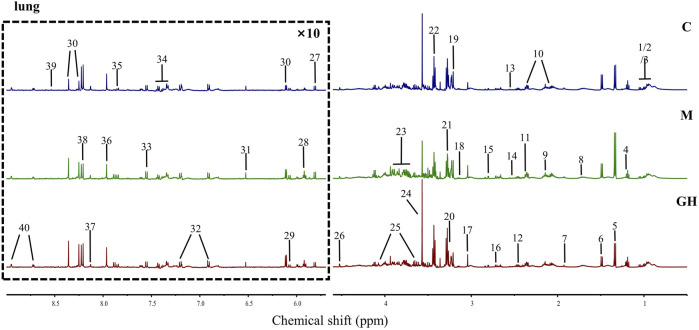
^1^H-NMR map and identified metabolites of rat lung tissue. The metabolites in the lung tissue in the picture were as follows: 1 Isoleucine (Ile), 2 Leucine (Leu), 3 Valine (Val), 4 3-Hydroxybutyrate (3-HB), 5 Lactate (Lac), 6 Alanine (Ala), 7 Acetate (AC), 8 Lysine (Lys), 9 Methionine (Met), 10 Glutamate (Glu), 11 Pyruvate (Pyru), 12 Glutamine (Gln), 13 Glutathione (GSH), 14 Isocitrate, 15 Aspartate (Asp), 16 dimethylamine (Dma), 17 Creatine/Phosphocreatine (CP), 18 Ethanolamine (MEA), 19 Choline, 20 Betaine (Bet), 21 Trimethylamine N-oxide (TMAO), 22 Taurine (Tau), 23 Glucose (Glc), 24 Glycine (Gly), 25 Myo-inositol, 26 Ascorbate, 27 Uracil (U), 28 Uridine, 29 Adenosine, 30 Inosine, 31 Fumarate (Fum), 32 Tyrosine (Tyr), 33 Tryptophan (Try), 34 Phenylalanine (Phe), 35 Cytidine, 36 Xanthine (Xan), 37 Carnosine (Carn), 38 Hypoxanthine (Hyp), 39 Formate (FA), 40 Nicotinamide/Nicotinurate (vpp).

Metabonomics can be used to study the type and quantity of endogenous metabolites and their changes under the influence of internal and external factors (Lihua et al., 2017). In this experiment, we used the ^1^H-NMR spectroscopy to examine the metabolic profiles of the lungs. Overall, 40 small molecular metabolites were identified in the lung tissues, which were mainly lipid, amino acids, and glucose metabolites. The ^1^H-NMR map of the lung tissue is shown in Figure 2, and atlas attribution data are shown in Supplementary Table S3.

**FIGURE 3:**
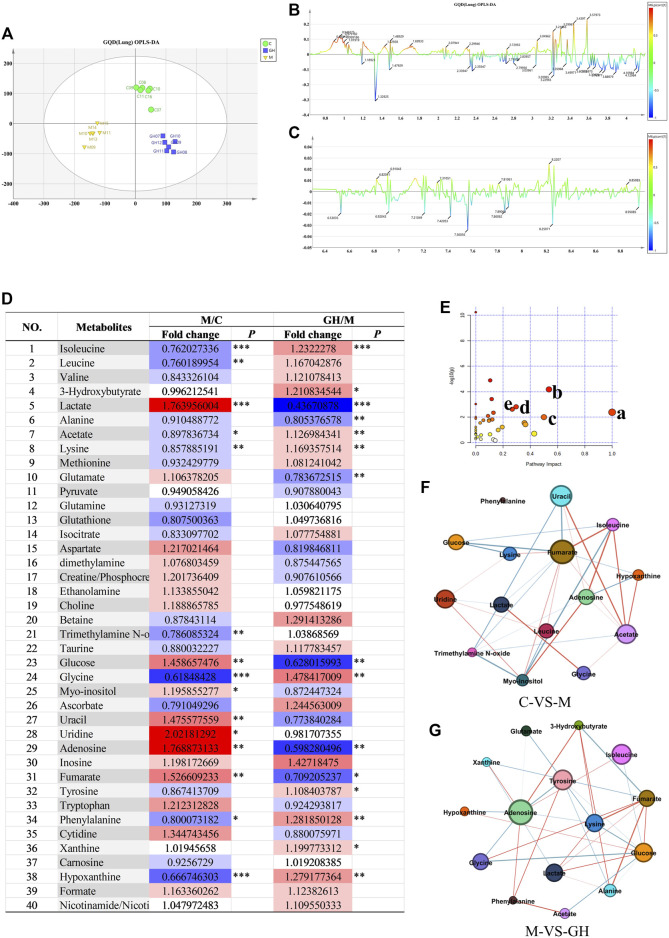
Analysis of lung tissue metabolites of GQD on ALI induced by LPS in rats. **(A)** OPLS-DA analysis, the abscissa is the main component T1, and the ordinate is the main component T2. Blue is GQD treatment group, yellow is model group, green is blank group. **(B)** OPLS-DA load diagram of high field region, **(C)** OPLS-DA load diagram in low Field region, the horizontal axis is the chemical shift, and the vertical axis is the *P* (corr) value of the difference between groups. The greater the absolute value of the vertical axis, the greater the difference between groups of metabolites at the chemical shift. **(D)** Univariate analysis Color Table, the color coding is performed according to the change in magnification (FC). The darker the color, the higher the significance, the darker the red, the higher the positive magnification of the difference between the two groups, and the darker the blue, the higher the reverse magnification of the difference between the two groups. The t-test or non-parametric Mann-Whitney test was performed, and the *p* values were corrected by the Benjamini-Hochberg method, *p < 0.05, **p < 0.01, ***p < 0.001. **(E)** Bubble diagram of metabolic pathway, the bubble area is proportional to the influence of the pathway, and the color ranges from red to white, which represents the maximum significance to the minimum significance. **(F)** Network diagram of small molecular metabolites in C-VS-M group. **(G)** Network diagram of small molecular metabolites in M-VS-GH group. The metabolites with Pearson correlation coefficient higher than the threshold are connected by solid lines, which are color-coded according to the value of the coefficient (warm color represents positive correlation and cool color represents negative correlation).

Orthogonal partial least squares discriminant analysis (OPLS-DA) was used for multivariate analysis of metabolic profile data, and the results showed considerable differences in the metabolites detected in the lung tissues between the treatment groups and slight differences within groups (Figure 3A), suggesting that the metabolic data were stable and reliable. PLS-DA load diagram showed that there were more significant changes in small molecular metabolites (Figures 3B,C). Furthermore, univariate analysis of identified metabolites showed that LPS-induced ALI significantly affected the concentration of metabolites in the lung tissues of the experimental rats (Figure 3D). However, GQD treatment reversed these effects to varying degrees in the different treatment groups.

To further identify ALI-associated metabolic pathways in rat lung tissue, pathway analysis and visualization was performed using MetbraAnalyst 5.0 software. According to Figure 3E, major significant ALI-associated metabolic pathways were: 1) phenylalanine, tyrosine, and tryptophan metabolic pathways; 2) alanine, aspartic acid, and glutamate metabolic pathways; 3) glutamine and glutamate metabolism; 4) glycine, serine, and threonine metabolic pathways; and 5) ketone metabolic pathway. These pathways are mainly related to energy and amino acid metabolism. The results indicated that LPS-induced ALI can cause energy and amino acid metabolism disorders and that GQD treatment can reverse these effects through metabolic remodeling.

Finally, the relationships between the significant metabolites (Figures 3F,G) were determined using Person’s correlation analysis, and the results showed that the core metabolites were glycine and lysine. Heat map of the metabolites showed that LPS-induced ALI caused a significant decrease in the concentration of glycine and lysine (Figure 3D); however, GQD treatment reversed this effect. The above results suggest that glycine and lysine play important roles in ALI and are potential small molecular metabolic markers for the clinical diagnosis and treatment of ALI.

### 3.3 Transcriptomics Analysis Results of Gegen Qinlian Decoction-Treated LPS-Induced Acute Lung Injury Rats

**FIGURE 4:**
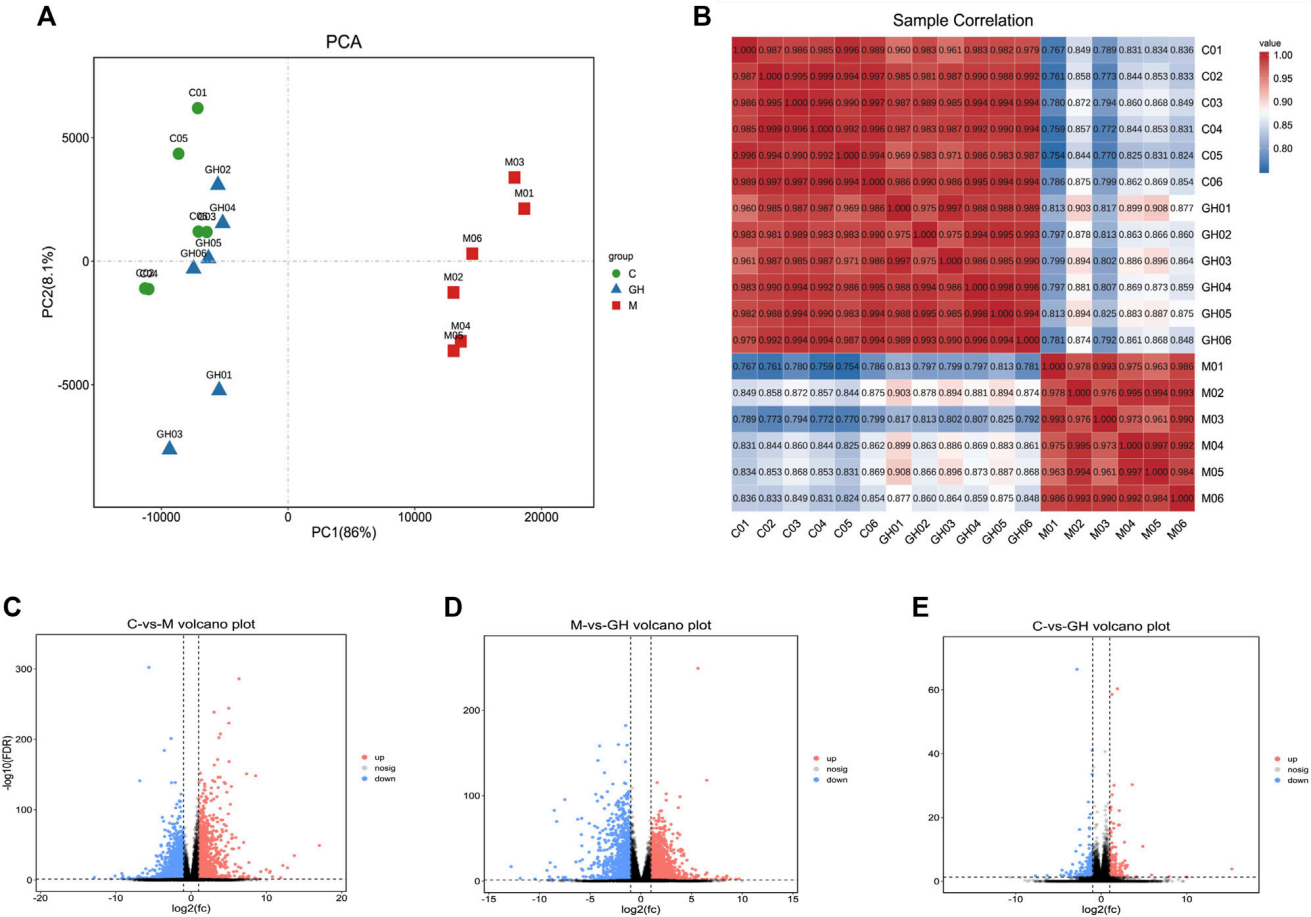
Analytical diagram of rat lung tissue samples. **(A)** The first principal component is represented by the PC1 coordinate. The second principal component is represented by PC2 coordinates. The contribution of the principal component to the sample difference is expressed as a percentage. Groups are represented by different colored graphs. **(B)** Sample correlation heat map. The abscissa and ordinate in the figure are for each sample, and the color depth indicates the correlation coefficient of the two samples. The closer to red or blue, the greater the correlation, and the closer to white, the lower the correlation. **(C)** Comparison between blank group and model group, **(D)** Comparison between model group and administration group, **(E)** Comparison between blank group and administration group. The Abscissa represents the logarithm of the difference multiple between the two groups, the ordinate indicates the negative log_10_ value of the FDR of the difference between the two groups, the red (group_2 up-regulated relative to group_1, expression) and blue (down-regulated expression) points indicate that there is a difference in gene expression (the criterion is FDR <0.05, and the difference multiple is more than twice), and the black point shows no difference.

Transcriptomic analysis is used to examine the expression profiles of genes under different treatment conditions (Li-Hua et al., 2015). Similar to the results in Figures 4A,B, the gene expression profiles of the lung tissues of rats in the control and GQD groups were similar and highly correlated. In contrast, the gene expression profiles of the lung tissues of rats in the control and treatment groups were significantly different from those of LPS-induced ALI rat models with low correlation. Overall, the difference in the gene expression profiles of the lung tissues between treatment groups was large, while the difference within groups was small, suggesting that the data was stable and reliable.

Based on the significantly different genes of each group, we drew a volcano map of the differences between the groups. (Figures 4C–E). A comparison of the data of the three volcanic maps showed that the gene expression profiles of C vs. M group (Figure 4C) and the M vs. GH group (Figure 4D) were significantly different. The above results indicated that GQD treatment effectively regulated the gene expression profile of LPS-induced ALI rats.

**FIGURE 5:**
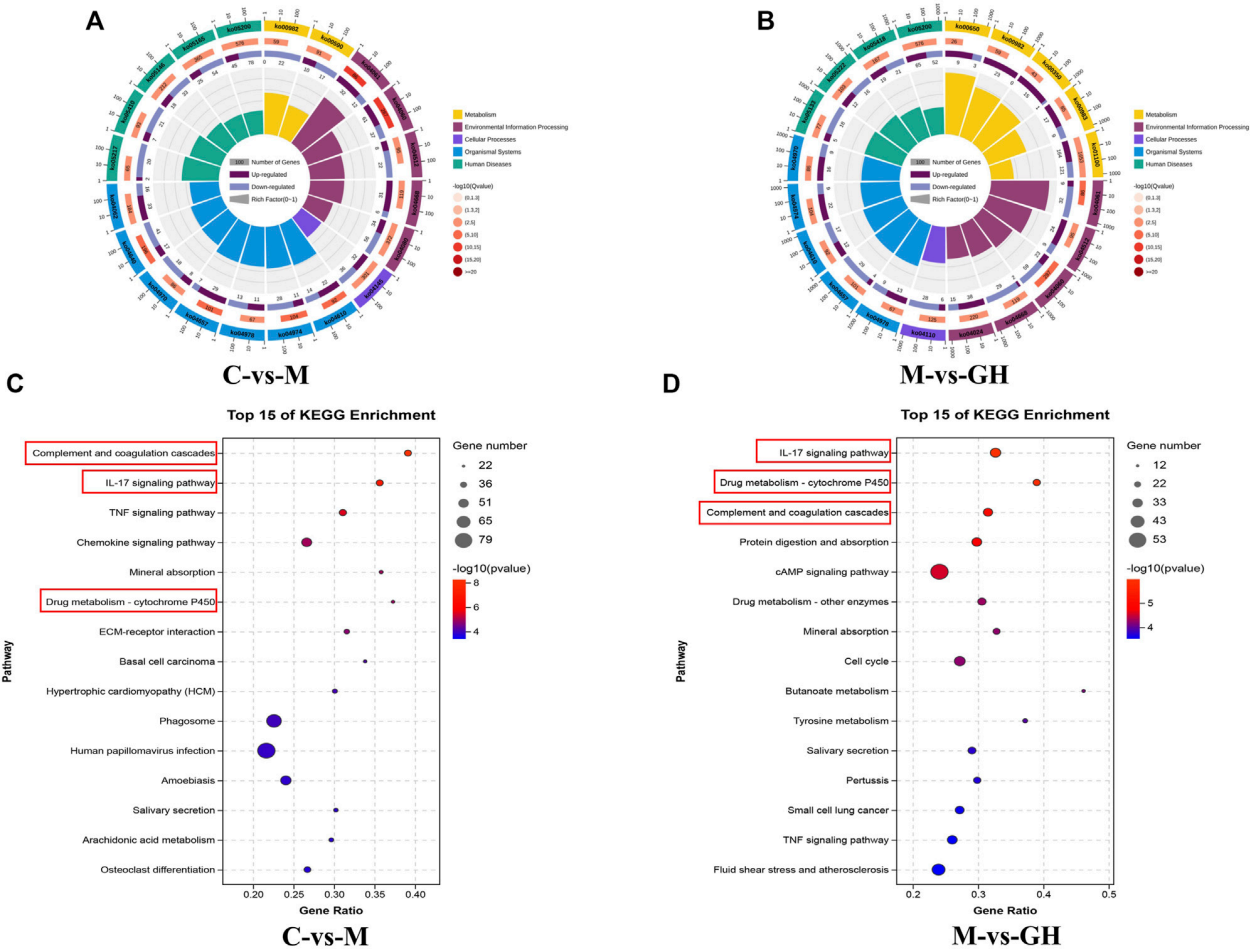
KEGG enrichment circle diagram and bubble diagram. **(A)** C-VS-M enrichment cycle diagram. **(B)** M-VS-GH enrichment circle diagram. The first circle is the pathway of the top 20 enrichment, and the coordinate ruler of the number of genes is outside the circle. Different colors represent different KEGG classifications. The second circle is the number of the pathway in the background gene and the Q value. The more genes, the longer the bar, and the smaller the Q value, the redder the color. The third circle is a bar chart of the proportion of up-regulated genes, with dark purple representing the proportion of up-regulated genes and light purple representing the proportion of down-regulated genes. The specific value is shown below. The fourth circle is the Rich Factor value of each pathway, the background grid line, each grid represents 0.1. **(C)** C-VS-M Bubble chart, **(D)** M-VS-GH Bubble chart. The ordinate is pathway, and the abscissa is the enrichment factor, the size indicates the quantity, the redder the color, the smaller the Q value, the higher the significance.

The KEGG enrichment circle diagram and bubble diagram are shown in Figure 5. Compared with Figure 5A, the yellow plate (metabolism class) in Figure 5B increased significantly, while the proportion of other plates did not change significantly. The results indicated that genes in the GQD treated groups were mostly enriched in metabolic pathways. Compared with Figures 5C,D, immune-related pathways, including “Complement and coagulation cascades,” “IL-17 signaling pathway,” and “Drug metabolism-cytochrome P450” were significantly enriched in C vs. M group and M vs. GH group, indicating that GQD can treat ALI by regulating immune-related pathways.

### 3.4 Gegen Qinlian Decoction Inhibits Immune Inflammatory Response in LPS-Induced Acute Lung Injury Rats

**FIGURE 6:**
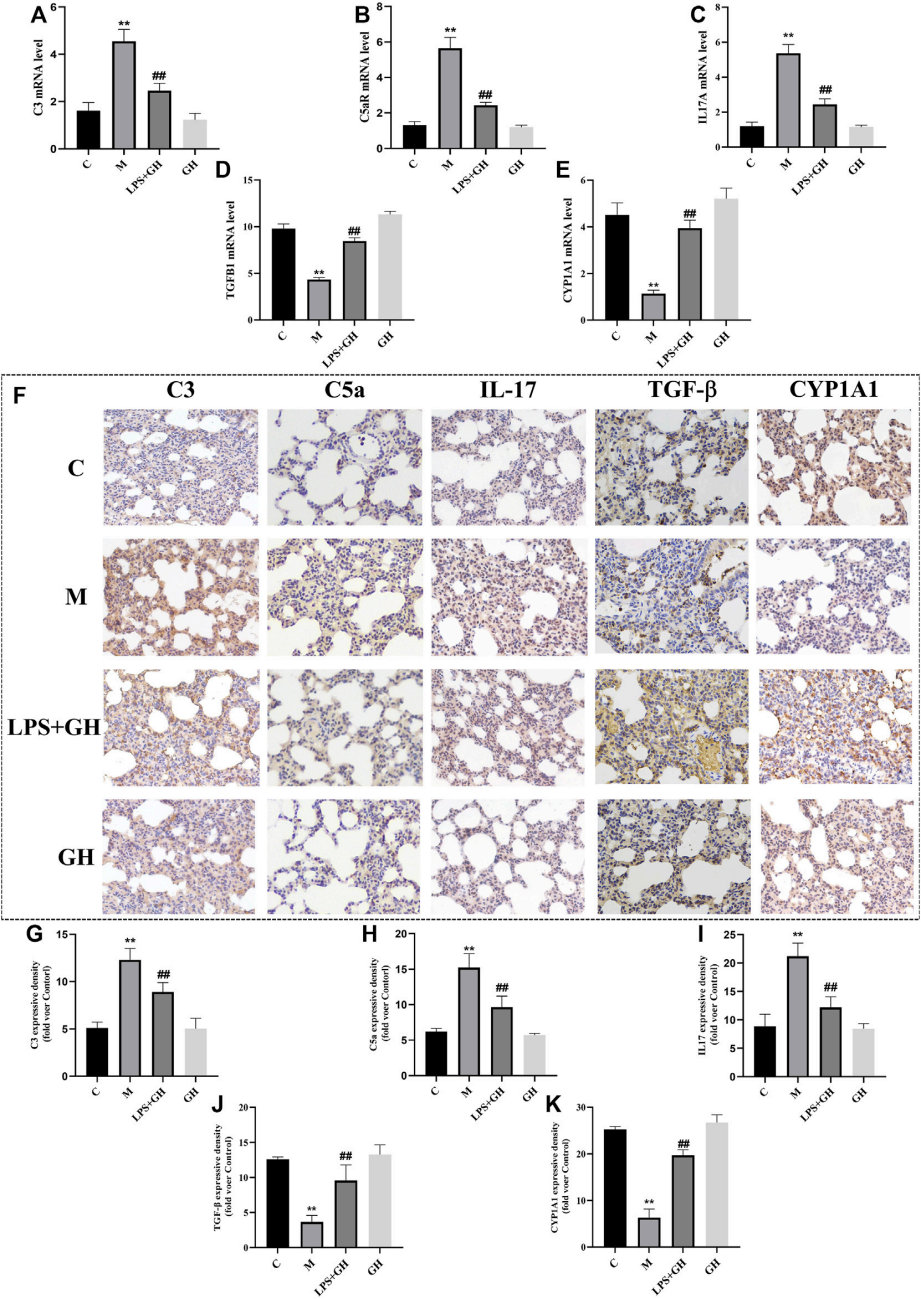
Immunohistochemistry and expression of key targets (*n* = 6). **(A)** The expression level of C3 mRNA. **(B)** The expression level of C5aR mRNA. **(C)** The expression level of IL-17A mRNA. **(D)** The expression level of TGF-β1 mRNA. **(E)** The expression level of CYP1A1 mRNA. **(F)** Immunohistochemical results of each group (Magnification ×400). **(G)** Expression of C3 protein. **(H)** Expression of C5a protein. **(I)** Expression of IL-17 protein. **(J)** Expression of TGF-β protein. **(K)** Expression of CYP1A1 protein. Compared with the control group, **p < 0.01. Compare with model group, ^##^ *p <* 0.01.

To verify the role of complement and coagulation cascades, IL-17 signaling pathway, and drug metabolism-cytochrome P450 pathways in immuno-inflammatory response, the targeted substances in these pathways were identified by RT-qPCR and immunohistochemical analysis. C3 is the hub of the complement activation pathway, which can trigger adaptive immune regulation in the body (Sun et al., 2011). Among the substances activated is C5a, an allergic toxin, which functions as an inflammatory mediator and produces biological effects through its receptor C5aR. Compared with the control group, LPS-induced ALI significantly upregulated the expression of C3 and C5a at the mRNA and protein levels in the experimental rats; however, GQD treatment significantly reversed this effect (Figures 6A,B,F–H).

IL-17 is the main effector of Th17 cells and is a proinflammatory cytokine. TGF-β is an important effector factor secreted by Treg cells. When the immune system is not activated, Tregs can secrete TGF-β normally to inhibit inflammation and prevent the occurrence of autoimmune diseases (Gap, 2018). Compared with the control group, LPS-induced ALI significantly increased the expression of IL-17 and decreased the expression of TGF-β at the both the mRNA and protein levels (Figures 6C,D,F,I,J); however, GQD treatment significantly reversed the effects of ALI. These results suggest that GQD can effectively inhibit the secretion of IL-17 by Th17 cells and promote the expression of TGF-β, thus regulating the balance of Th17/Treg cells to reduce inflammatory reactions associated with ALI.

As an important metabolic enzyme of the P450 family, CYP1A1 is closely related to inflammation. Studies (Lingappan et al., 2011) have shown that CYP1A1 activation may reduce lung injury by reducing lipid peroxides concentration in the body. Compared with the control group, LPS-induced ALI significantly decreased the expression of CYP1A1 at the mRNA and protein levels (Figures 6E,F,K); however, GQD treatment significantly reversed the effects of ALI. This indicated that GQD exerted anti-ALI effect by promoting CYP1A1 activation. Overall, GQD exerted anti-ALI effect by regulating three pathways mentioned above.

### 3.5 Effect of Gegen Qinlian Decoction on the Intestinal Microflora of LPS-Induced Acute Lung Injury Rats

**FIGURE 7:**
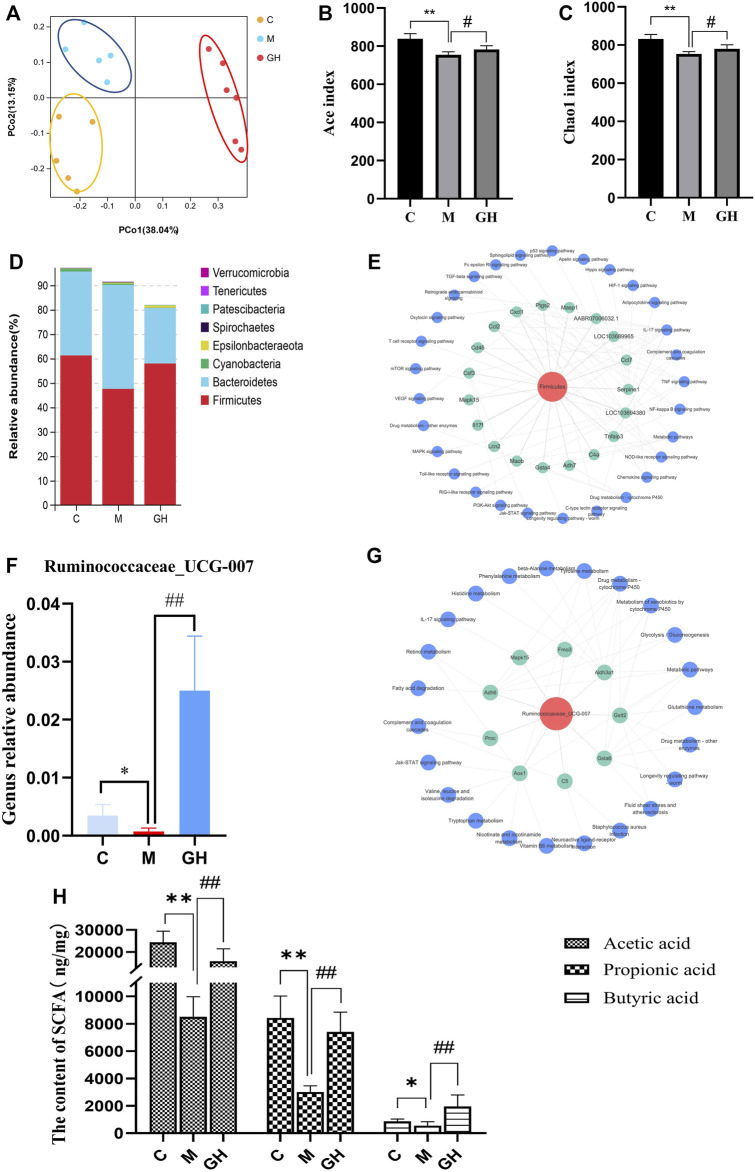
Effect of GQD on coliform bacteria in ALI rats induced by intratracheal injection of LPS. **(A)** PCoA analysis of intestinal flora in different groups of rats. Each point in the figure represents the sample, the closer the point distance on the plane, the more similar the structure of the color of the sample. **(B,C)** Alpha diversity analysis of gut bacterial richness from different mouse groups. **(D)** Distribution map of phylum level of intestinal flora in each group of rats. The vertical coordinate indicates the relative abundance of the species, the Abscissa indicates the grouping, C is the blank group, M is the model group, and GH is the GQD administration group. **(E)** Firmicutes-gene-pathway network diagram. The red in the picture is Firmicutes, the green is the gene, and the blue is the pathway. **(F)** The relative abundance of Ruminococcaceae_UCG_007 in each group. **(G)** Ruminococcaceae_UCG_007-Gene pathway network diagram. In the figure, the red is Ruminococcaceae_UCG_007, the green is the gene, and the blue is the pathway. **(H)** The content of short-chain fatty acids (acetic acid, propionic acid and butyric acid) in fecal samples of rats in each group. Compared with the control group, *p < 0.05, **p < 0.01; compared with the model group, ^#^ *p <* 0.05, ^##^ *p <* 0.01, *n* = 6.

The lung-gut axis theory states that alterations in intestinal microbiota community may have considerable effects on lung disease, indicating that the progression of lung diseases may depend on intestinal health (Chang et al., 2021). Therefore, the effect of GQD on the intestinal microbiota of LPS-induced ALI rats was examined in this study. Principal coordinate analysis was performed on the diversity data of microbiota of rats in each group. The results were shown in Figure 7A, there were significant differences in the microflora characteristics among the blank group, model group and GQD administration group, and GQD had a great influence on the microflora characteristics. Consistently, α-diversity analysis revealed that GQD prevented LPS-induced decrease in bacterial richness, as indicated by Ace and Chao1 indices (Figures 7B,C). In this study, we found that the intestinal flora of ALI rats induced by tracheal instillation of LPS were mainly concentrated in the changes of Firmicutes and Bacteroidetes. The relative abundance results were shown in Figure 7D. We found that the influence of this study on intestinal flora mainly focused on the changes of Firmicutes and Bacteroidetes. Compared with control group, the relative abundance of Firmicutes in model group decreased, while the relative abundance of Bacteroidetes increased. After the intervention of GQD, the relative abundance of Firmicutes and Bacteroides were reverted. To investigate whether Firmicutes are associated with the three signaling pathways identified by KEGG analysis of the transcriptome, we performed a correlation analysis of the 16s rDNA sequencing data and transcriptome data. Results showed that Firmicutes was associated with the three pathways, most closely associated with the IL-17 signaling and complement signaling pathways (Figure 7E). Additionally, Firmicutes were associated with other immunoinflammatory pathways, such as TNF and T cell receptor signaling pathways. These results indicated that Firmicutes may play important roles in the regulation of immunity and inflammation.

Among Firmicutes, the effect of GQD on the abundance of the genus Ruminococcaceae_UCG_007 was examined. Ruminococcaceae_UCG_007 is involved in butyric acid production (Yangyang, 2020). Interestingly, the change trend of its relative abundance was consistent with that of Firmicutes (Figure 7F). LPS-induced ALI significantly decreased the abundance of Ruminococcaceae_UCG_007; however, this was reversed by GQD treatment. Similarly, Ruminococcaceae_UCG_007 was associated with the three immune pathways (Figure 7G). Furthermore, Ruminococcaceae_UCG_007 was closely related to the amino acid metabolism pathway, which was consistent with the metabolomics results. The results indicate that Ruminococcaceae_UCG_007 may be the primary intestinal bacteria genus of GQD in treating ALI.

Additionally, analysis of SCFAs contents of the fecal samples collected from the rats showed that LPS-induced ALI significantly decreased the acetic, propionic, and butyric acid content of the fecal samples. However, GQD treatment reversed these effects to varying degrees (Figure 7H). In conclusion, GQD affected the generation of intestinal metabolites such as short-chain fatty acids by regulating the abundance of Firmicutes, thus improving the immune function of the body.

## 4 Discussion

Acute lung injury is a severe respiratory critical disease, with a mortality rate as high as 40% (Sweatt and Levitt, 2014; Butt et al., 2016). Presently, there is no effective drug treatment for ALI symptoms. GQD is a classic prescription in traditional Chinese medicine for the treatment of intestinal and pulmonary inflammatory diseases (Li et al., 2021), and is composed of *Puerariae lobatae radix*, *Scutellariae radix*, *Coptidis rhizoma*, and *Glycyrrhizae radix et rhizoma*. Existing studies have shown that GQD is effective against ALI, but the mechanism of its action is not clear (Jinfu et al., 2015). To better study the anti-ALI effect of GQD, we conducted a comprehensive study on LPS-induced ALI mice model (Ding et al., 2019). Among them, LPS-induced ALI model is recognized as an animal model with good reproducibility and highly similar to clinicopathological changes. According to the relevant reviews and descriptions included in *Am J Physiol Lung Cell Mol Physiol*, a classic journal in the field of breathing in the United States (Matute-Bello et al., 2008). There is a certain correlation between different ways of LPS induction and the severity of ALI in clinic. For instance, intratracheal injection or nasal infusion of LPS can directly induce pulmonary neutrophils and upregulate inflammatory factors in pulmonary ALI, which could simulate initial symptoms of ALI inflammation (There is no apoptosis phenomenon, which has been verified by experiments. The results are shown in Supplementary Figure S1). However, intraperitoneal injection of LPS could induce systemic septic ALI, which could simulate a more severe clinical stage. In a previous study using mice subjected to intraperitoneal injection of LPS, we found that GQD exerted its protective effect against ALI by activating the PI3K/Akt cell survival pathway (Ding et al., 2020). However, in the present study, using rats subjected to intratracheal injection of LPS, we found that GQD exerted its protective effect against ALI by regulating immune-related pathways. In short, different pathways of LPS modeling will lead to different pathogenesis and different pathological phenomena. Because GQD has the characteristics of multiple components, multiple targets, and multiple action pathways, it can improve ALI through different mechanisms in accordance with the pathological characteristics of ALI at different stages.

In the present study, to study the therapeutic effect of GQD on ALI rats induced by intratracheal injection, we conducted an analysis of conventional indicators of lung tissue injury. In the pharmacodynamic experimental analysis, we examined the effect of GQD on the wet/dry weight ratio of the lungs and BALF protein content, and found that GQD significantly decreased the wet/dry weight ratio of the lungs and the BALF protein content. The results showed that GQD was effective in maintaining alveolar-vascular barrier integrity, inhibiting pulmonary edema, and protecting against LPS-associated cell injury. Additionally, GQD reduced the respiratory frequency, increased the respiratory cycle and respiratory depth, and improved the gas exchange ability of the ALI rat model. Previous research has confirmed that TNF-α, IL-1β, and IL-6 are important cytokines in the development of ALI, and can induce neutrophil activation and polymerization to inflammatory sites (Tai et al., 2017). In the present study, GQD treatment significantly reversed ALI-associated increase in BALF and serum TNF-α, IL-1β, and IL-6 concentrations, confirming that GQD can effectively regulate the inflammatory responses associated with LPS-induced ALI.

Furthermore, metabolomic analysis was performed to examine the metabolic profile of the lung tissues of the rat, and we found that the metabolic profiles of the lung tissues were significantly affected by the treatments, and that the differential metabolites between treatment groups were mainly involved in energy and amino acid metabolism. Additionally, bioinformatics analysis showed that glycine and lysine were the main metabolites through which GQD exerted its effect against ALI. It has been found that glycine preconditioning can reduce LPS-induced collagen deposition, alveolar cell apoptosis, expression of inflammatory cytokines and chemokines, and neutrophil and macrophage accumulation in rat lung tissue, thus improving alveoli integrity and function (Ma et al., 2019). Lysine is a basic amino acid that promotes human development and enhances immune function (Li-Qing et al., 2014). Some studies have confirmed that lysine can reduce ALI-associated inflammatory response and protect the lungs (Zhang Y. et al., 2019). A recent study published in Nature Communications showed metabolic remodeling and inflammatory response are closely related in the serum of ALI patients (Xiao et al., 2021). However, regulating amino acid metabolism could significantly ameliorate excessive immune inflammatory response. The findings of the present study are similar to those of the above referenced studies, suggesting that GQD can regulate amino acid metabolism and improve the inflammatory response in ALI rats.

KEGG enrichment analysis in transcriptomics showed that immune-related pathways, including complement and coagulation cascades, IL17 signaling pathway, and drug metabolism-cytochrome P450, were significantly enriched after GQD treatment, suggesting that GQD exerted anti-ALI effects by regulating immune-related pathways. The complement signaling pathway is an important part of innate immunity and plays a key role in the inflammatory response. Recent studies (Bosmann and Ward, 2012) have shown that C3 is the hub of the complement activation pathway, which not only participates in natural defense, but also triggers adaptive immune regulation, which to a large extent can cause the occurrence and development of acute or chronic inflammatory diseases. A large number of active fragments are produced during complement activation, most of which function as inflammatory mediators, among which C5a has the strongest effect (Carvelli et al., 2020). The findings of the present study showed that GQD inhibited the activity of complement and complement fragments, such as C3 and C5a. According to the latest research report (Quanming et al., 2020), the BDB001 injection developed by German InflaRx company for COVID-19. It is a monoclonal antibody drug against human C5a molecule, which can specifically bind C5a, thus blocking the binding of C5a to the receptor, inhibiting the inflammatory cascade reaction and improving the lung injury caused by virus-induced complement overactivation. The mechanism of BDB001 is consistent with that of GQD, indicating that GQD can be used as a potential drug in treating pulmonary inflammatory diseases. IL-17 is a major effector of Th17 cells, which are cytokines that cause inflammation (Miossec and Kolls, 2012). Treg cells are T cells that regulate immunosuppression in the body, which can reduce tissue injury by controlling the intensity of immune response (Wang and Qian, 2010). During ALI, the balance between Th17 and Treg cells is disrupted, resulting in inflammation. When the balance is more weighted towards Th17 cells, Th17 cells can recruit the infiltration of inflammatory cells by releasing a large number of pro-inflammatory factors such as IL-17 and at the same time antagonize the number of Treg cells, thus further aggravating the inflammatory injury (Gap, 2018). GQD can reduce the ALI-associated inflammatory response by inhibiting the activity of Th17 cells and promoting the expression of Treg cells to restore the balance between Th17 and Treg. In addition, in the screening of pathways, we found that the Drug metabolism-cytochrome P450 pathway was also significant. Among which CYP1A1 is involved in inflammation (van Schaik, 2008). Previous studies have shown that CYP1A1 may reduce lung injury by reducing the levels of lipid peroxides in the body (Lingappan et al., 2013). In the present study, GQD exerted anti-ALI effect by increasing the expression of CYP1A1. Among the differential genes, most CYP450 enzymes were upregulated after GQD treatment, suggesting that these CYP450 enzymes can promote the metabolism of GQD in the body, thus accelerating its functions in lungs. Overall, GQD exhibited anti-ALI effect by regulating multiple immune-related pathways.

Intestinal microbiota composition has been linked to immune responses and inflammation, and may play important roles in the pathological immune response of the intestine and lung (Fan and Pedersen, 2020). Intestinal microbiota indirectly regulates systemic immunity by regulating T cell populations and metabolizing short-chain fatty acids (Blander et al., 2017). Existing studies have confirmed that GQD can play an immunomodulatory role by regulating the composition of intestinal microbiota to improve symptoms of some intestinal diseases (Yang, 2019; Yang, 2020). According to the theory of gut-lung axis, we used 16s rDNA sequencing to explore whether GQD can improve ALI by affecting intestinal flora. According to the analysis of the overall results of PCoA, GQD has an effect on the structural characteristics of intestinal flora. From the results of α diversity analysis, it can be concluded that LPS treatment decreased the richness of intestinal microbiota in rats, and GQD could inhibit this trend, which suggested that GQD has a certain regulatory effect on intestinal microbiota. In this study, early symptoms of ALI were induced by intratracheal injection of LPS in rats, and the influences on intestinal flora of rats were mainly the changes of Firmicutes and Bacteroidetes. Existing studies have shown that bacteria of Firmicutes are involved in the fermentation of dietary components, producing metabolites such as short-chain fatty acids (SCFAs) (Zhang X. et al., 2019). SCFAs are important in maintaining intestinal homeostasis, and also play important roles in immune response. SCFAs can reduce colon oxidative stress, inhibit pathogen growth, regulate inflammation-related signaling pathways maintain Th17/Treg cells balance, maintain the intestinal mucosal barrier, and are involved in inflammatory response (Sun et al., 2016; Shuang et al., 2019; Kexin et al., 2020). Therefore, a decrease in the abundance of Firmicutes can lead to an imbalance in intestinal microbiota, which can affect intestinal metabolism, immune responses, consequently, leading disease emergence. In the present study, correlation analysis of 16s rDNA sequencing data and transcriptomeic data showed that Firmicutes was closely related to IL-17 signaling pathway and complement signaling pathway. This finding indicated that Firmicutes may play important roles in inflammation and immune regulation, and that GQD may treat ALI by regulating the abundance of Firmicutes. At the genus level, the effect of GQD on the relative abundance of Ruminococcaceae_UCG_007, a butyric acid producing genus (Yangyang, 2020). Results of the joint analysis showed that Ruminococcaceae_UCG_007 was closely related to amino acids metabolic pathway, which was consistent with the result of the metabolomic analysis. These findings indicated that Ruminococcaceae_UCG_007 may be an important genus of intestinal microbiota and may play an important role in intestinal microbiota metabolism. Furthermore, GQD treatment upregulated the SCFAs content (acetic, propionic, and butyric acid) of the fecal samples of the rats. Overall, it can be concluded that GQD increased the SCFAs content by regulating Firmicutes abundance, specifically Ruminococcaceae_UCG_007 abundance, so as to regulate the immune function of the body.

In conclusion, intratracheal instillation of LPS was used to induce the initial stage of ALI. From the point of view of immunity, GQD played a role in improving ALI by regulating immune function. GQD can control the occurrence and development of ALI by improving the metabolic remodeling of the body, affecting several immune pathways (Inhibition of complement pathway activation, regulation of Th17/Treg balance) and by regulating intestinal microbiota. Overall, the findings of the study provide a solid scientific research foundation for promoting the clinical use of GQD in treating ALI.

## Data Availability

The datasets presented in this study can be found in online repositories. The names of the repository/repositories and accession number(s) can be found below: https://www.ncbi.nlm.nih.gov/sra; SRR17814908 to SRR17814919; SRR17818112 to SRR17818129.

## References

[B1] Blander J. M. Longman R. S. Iliev I. D. Sonnenberg G. F. Artis D. (2017). Regulation of Inflammation by Microbiota Interactions with the Host. Nat. Immunol. 18, 851–860. 10.1038/ni.3780 28722709 PMC5800875

[B2] Bosmann M. Ward P. A. (2012). Role of C3, C5 and Anaphylatoxin Receptors in Acute Lung Injury and in Sepsis. Adv. Exp. Med. Biol. 946, 147–159. 10.1007/978-1-4614-0106-3_9 21948367 PMC3372066

[B3] Butt Y. Kurdowska A. Allen T. C. (2016). Acute Lung Injury: A Clinical and Molecular Review. Arch. Pathol. Lab. Med. 140 (4), 345–350. 10.5858/arpa.2015-0519-RA 27028393

[B4] Carvelli J. Demaria O. Vély F. Batista L. Chouaki Benmansour N. Fares J. (2020). Association of COVID-19 Inflammation with Activation of the C5a-C5aR1 axis. Nature 588 (7836), 146–150. 10.1038/s41586-020-2600-6 32726800 PMC7116884

[B5] Chang L. Xiong-Wei L. Jia-Xin L. (2021). Effect of Lonicera japonica Thunb and Lonicera Hypoglauca on Gut Microbiota in Rats with Acute Lung Injury Based on 16S rRNA Gene Sequencing[J]. Chin. J. Microecology 33 (02), 130–137. 10.13381/j.cnki.cjm.202102002

[B6] Dan R. L. Rudensky A. Y. (2010). Th17 and Regulatory T Cells in Mediating and Restraining Inflammation[J]. Cell. Physiol. Biochem. 140 (6), 845–858. 10.1016/j.cell.2010.02.021 20303875

[B7] Dang A. T. Marsland B. J. (2019). Microbes, Metabolites, and the Gut-Lung axis. Mucosal Immunol. 12, 843–850. 10.1038/s41385-019-0160-6 30976087

[B8] Dayong X. (2018). Clinical Application of Maxingshigan Decoction Combined with Gegenqinlian Decoction in Conventional Treatment of Community-Acquired pneumonia[D]. Beijing University of Chinese Medicine.

[B9] Ding Z. Zhong R. Xia T. Yang Y. Xing N. Wang W. (2019). Advances in Research into the Mechanisms of Chinese Materia Medica against Acute Lung Injury. Biomed. Pharmacother. 122, 109706. 10.1016/j.biopha.2019.109706 31918277

[B10] Ding Z. Zhong R. Yang Y. Xia T. Wang W. Wang Y. (2020). Systems Pharmacology Reveals the Mechanism of Activity of Ge-Gen-Qin-Lian Decoction against LPS-Induced Acute Lung Injury: A Novel Strategy for Exploring Active Components and Effective Mechanism of TCM Formulae. Pharmacol. Res. 156, 104759. 10.1016/j.phrs.2020.104759 32200026

[B11] Fan Y. Pedersen O. (2020). Gut Microbiota in Human Metabolic Health and Disease. Nat. Rev. Microbiol. 19, 55–71. 10.1038/s41579-020-0433-9 32887946

[B12] Gai-Jun Z. Jing M. Li-Ying G. (2021). Application of Multi-Omics Combination in Mechanism Studies of Traditional Chinese Medicine[J]. Chin. Traditional Herb. Drugs 52 (10), 3112–3120.

[B13] Gai-Jun Z. Xi-Yu L. Lu-Lu J. (2021). Research Progress on the Relationship between the Balance of Treg/Th17 and in Sepsis[J]. Chin. J. Immunol., 1–14. 10.3969/j.issn.1000-484X.2021.19.020

[B14] Gap L. (2018). The Balance of Th17 versus Treg Cells in Autoimmunity[J]. Int. J. Mol. Sci. 19 (3), 730. 10.3390/ijms19030730 PMC5877591 29510522

[B15] He Y. Wen Q. Yao F. Xu D. Huang Y. Wang J. (2017). Gut-lung axis: The Microbial Contributions and Clinical Implications. Crit. Rev. Microbiol. 43, 81–95. 10.1080/1040841X.2016.1176988 27781554

[B16] Jinfu Y. Xiangze F. Ziyao Y. (2015). Protective Mechanism of Gegen Qinlian Decoction on Endotoxin-Induced Acute Lung Injury in Mice[J]. Chin. J. Gerontology 35 (07), 1899–1900.

[B17] Kexin W. Ning J. Aizhong Z. (2020). Host Intestinal Immune Regulation Mechanism Mediated by Short-Chain Fatty Acids[J]. Chin. J. Animal Nutr. 032 (004), 1544–1550. 10.3969/j.issn.1006-267x.2020.04.010

[B18] Kim Y. Y. Lee S. Kim M. J. Kang B. C. Dhakal H. Choi Y. A. (2017). Tyrosol Attenuates Lipopolysaccharide-Induced Acute Lung Injury by Inhibiting the Inflammatory Response and Maintaining the Alveolar Capillary Barrier. Food Chem. Toxicol. 109, 526–533. 10.1016/j.fct.2017.09.053 28974441

[B19] Li J. Lin X. Liu X. Ma Z. Li Y. (2020). Baicalin Regulates Treg/Th17 Cell Imbalance by Inhibiting Autophagy in Allergic Rhinitis. Mol. Immunol. 125, 162–171. 10.1016/j.molimm.2020.07.008 32688118

[B20] Li W. Ling P. Rong C. (2021). Clinical Application and Experimental Research Progress of Pueraria and Scutellaria and Coptis Decoction[J]. Henan Tradit. Chin. Med. 41 (04), 527–531. 10.16367/j.issn.1003-5028.2021.04.0122

[B21] Li-Hua X. Xing F. Ze-Jun L. (2015). Application of Transcriptomic Technologies in Hepatotoxicity Induced by Chinese Materia Medica[J]. Chin. Traditional Herb. Drugs 46 (10), 1536–1541.

[B22] Li-Qing H. Jiang-Hua G. Xin G. (2014). Metabonomics Study of the Rats Lung Tissue with Syndrome Suitable for Xiaoqinglong Decoction Based on NMR[J]. China J. Traditional Chin. Med. Pharm. 29 (07), 2338–2340.

[B23] Liang Q. Lin Q. Li Y. Luo W. Huang X. Jiang Y. (2020). Effect of SIS3 on Extracellular Matrix Remodeling and Repair in a Lipopolysaccharide-Induced ARDS Rat Model. J. Immunol. Res. 2020 (12), 6644687–6644713. 10.1155/2020/6644687 33294466 PMC7714568

[B24] Lihua M. Hongjing Y. Xiaoyan X. (2017). Advances in Metabonomics[J]. J. Mod. Med. Health 33 (17), 2636–2639.

[B25] Lin Y. Yang Y. (2018). MiR-24 Inhibits Inflammatory Responses in LPS-Induced Acute Lung Injury of Neonatal Rats through Targeting NLRP3[J]. Pathology, Res. Pract., 215, 683–688. 10.1016/j.prp.2018.12.028 30600184

[B26] Lingappan K. Jiang W. Wang L. Couroucli X. I. Barrios R. Moorthy B. (2013). Sex-specific Differences in Hyperoxic Lung Injury in Mice: Implications for Acute and Chronic Lung Disease in Humans. Toxicol. Appl. Pharmacol. 272 (2), 281–290. 10.1016/j.taap.2013.06.007 23792423 PMC4201582

[B27] Lingappan K. Jiang W. Wang L. Wang G. Couroucli X. I. Shivanna B. (2011). Mice Deficient in the Gene for Cytochrome P450 (CYP)1A1 Are More Susceptible Than Wild-type to Hyperoxic Lung Injury: Evidence for Protective Role of CYP1A1 against Oxidative Stress. Toxicol. Sci. 141 (1), 68–77. 10.1093/toxsci/kfu106 PMC4200035 24893714

[B28] Ma X. Zhang Y. Jiang D. Yang Y. Wu G. Wu Z. (2019). Protective Effects of Functional Amino Acids on Apoptosis, Inflammatory Response, and Pulmonary Fibrosis in Lipopolysaccharide-Challenged Mice. J. Agric. Food Chem. 67, 4915–4922. 10.1021/acs.jafc.9b00942 31001980

[B29] Markowiak-Kopeć P. Śliżewska K. (2020). The Effect of Probiotics on the Production of Short-Chain Fatty Acids by Human Intestinal Microbiome. Nutrients 12 (4), 1107. 10.3390/nu12041107 PMC7230973 32316181

[B30] Matute-Bello G. Frevert C. W. Martin T. R. (2008). Animal Models of Acute Lung Injury. Am. J. Physiol. Lung Cell Mol. Physiol. 295 (3), L379–L399. 10.1152/ajplung.00010.2008 18621912 PMC2536793

[B31] Ming J. Li-Yuan W. Jing J. (2016). Overview of Complement-Targeted Therapy[J]. Chin. J. Biochem. Pharm. 36 (12), 7–10+3.

[B32] Miossec P. Kolls J. K. (2012). Targeting IL-17 and TH17 Cells in Chronic Inflammation. Nat. Rev. Drug Discov. 11 (10), 763–776. 10.1038/nrd3794 23023676

[B33] Moni M. A. Liò P. (2015). How to Build Personalized Multi-Omics Comorbidity Profiles. Front. Cell Dev. Biol. 3, 28. 10.3389/fcell.2015.00028 26157799 PMC4478898

[B34] Qiong M. Jun M. Yi D. (2020). Application of Multi-Omics in Toxicity and Detoxification of Chinese Materia Medica[J]. Chin. Traditional Herb. Drugs 51 (12), 3117–3125.

[B35] Quanming Z. Haibo L. Hao Z. (2020). Current Status and Countermeasures for Development of Drugs to Treat Coronavirus Disease 2019[J]. J. Third Mil. Med. Univ. 42 (09), 861–866. 10.16016/j.1000-5404.202002233

[B36] Sadeghi A. Tahmasebi S. Mahmood A. Kuznetsova M. Valizadeh H. Taghizadieh A. (2020). Th17 and Treg Cells Function in SARS-CoV2 Patients Compared with Healthy Controls. J. Cell Physiol. 236, 2829–2839. 10.1002/jcp.30047 32926425

[B37] Shiheng W. (2020). Study on Clinical Literature of Gegen Qinlian Decoction for Enteritis Diseases Based on the Main Database[D]. China Academy of Chinese Medical Sciences.

[B38] Shuang L. A. Wen R. Qing W. Zhongxiang Z. Feifei N. Yajun F. (2019). Rhubarb Peony Decoction Ameliorates Ulcerative Colitis in Mice by Regulating Gut Microbiota to Restoring Th17/Treg Balance[J]. J. Ethnopharmacol. 231, 39–49. 10.1016/j.jep.2018.08.033 30170079

[B39] Sun M. Wu W. Liu Z. Cong Y. (2016). Microbiota Metabolite Short Chain Fatty Acids, GPCR, and Inflammatory Bowel Diseases. J. Gastroenterol. 52 (1), 1–8. 10.1007/s00535-016-1242-9 27448578 PMC5215992

[B40] Sun S. Wang H. Zhao G. An Y. Guo Y. Du L. (2011). Complement Inhibition Alleviates Paraquat-Induced Acute Lung Injury. Am. J. Respir. Cell Mol. Biol. 45 (4), 834–842. 10.1165/rcmb.2010-0444OC 21421909 PMC5460894

[B41] Sun S. W. Chen L. Zhou M. Wu J. H. Meng Z. J. Han H. L. (2019). BAMBI Regulates Macrophages Inducing the Differentiation of Treg through the TGF-β Pathway in Chronic Obstructive Pulmonary Disease. Respir. Res. 20 (1), 26. 10.1186/s12931-019-0988-z 30728014 PMC6364453

[B42] Sweatt A. J. Levitt J. E. (2014). Evolving Epidemiology and Definitions of the Acute Respiratory Distress Syndrome and Early Acute Lung Injury. Clin. Chest Med. 35 (4), 609–624. 10.1016/j.ccm.2014.08.002 25453413

[B43] Tai W. Xu Y. Ding J. Wu H. Du M. Qu X. (2017). Fibrocytes Ameliorate Acute Lung Injury by Decreasing Inflammatory Cytokine and Chemokine Levels and Reducing Neutrophil Accumulation in the Lung. Cell Physiol. Biochem. 44 (4), 1526–1536. 10.1159/000485647 29197869

[B44] van Schaik R. H. (2008). CYP450 Pharmacogenetics for Personalizing Cancer Therapy. Drug Resist Updat 11 (3), 77–98. 10.1016/j.drup.2008.03.002 18486526

[B45] Wang R. Xiao H. Guo R. Li Y. Shen B. (2015). The Role of C5a in Acute Lung Injury Induced by Highly Pathogenic Viral Infections. Emerg. Microbes Infect. 4 (5), e28. 10.1038/emi.2015.28 26060601 PMC4451266

[B46] Wang W-W. Qian S. (2010). Expression and Relationship of Th17 Cells and Treg Cells in Common Human Diseases[J]. Chin. J. Immunol. 26 (03), 284–288.

[B47] Wang Z-X. (2019). Astragaloside IV Regulates Autophagy and Apoptosis in PM2.5-induced Acute Lung Injury via AMPK/mTOR Pathway [D]. Chengdu University of TCM.

[B48] Xiao N. Nie M. Pang H. Wang B. Hu J. Meng X. (2021). Integrated Cytokine and Metabolite Analysis Reveals Immunometabolic Reprogramming in COVID-19 Patients with Therapeutic Implications. Nat. Commun. 12 (1), 1618. 10.1038/s41467-021-21907-9 33712622 PMC7955129

[B49] Yang C. (2019). Study on the Mechanism of Gegen Qinlian Decoction Based on Gut Microbiota in Model Rats with Acute Enteritis [D]. Shanghai University of Traditional Chinese Medicine.

[B50] Yang L. (2020). Effect of Gegen Qinlian Decoction on Gut Microbiota and Intestinal Barrier Function in Patients with Colorectal Cancer [D]. North China University of Science and Technology.

[B51] Yangyang X. (2020). The Changes and Mechanisms of Intestinal Microbiota and Sodium Butyrate in Acute Pancreatitis with Acute Respiratory Distress syndrome[D]. Peking Union Medical College.

[B52] Yi Z. Chen C. Jingchao S. (2021). Preparation of Rat Model of Acute Lung Injury and Comparison of Injury at Different Periods[J]. Acta Lab. Anim. Sci. Sin. 29 (01), 27–34. 10.3969/j.issn.1005-4847.2021.01.004

[B53] Yimin C. Jianping S. Shelan L. (2017). Correlation of Gut Microbiota and Respiratory Microbiota with Lung Diseases: Research Progress[J]. Chin. J. Microecology 29 (10), 1234–1241. 10.13381/j.cnki.cjm.201710028

[B54] Yun Z. Jia-Jia L. Jian-Yun W. (2020). Intestinal Microbiota-Lung axis and Lung Disease[J]. Chin. J. Veterinary Sci. 40 (02), 429–434. 10.16303/j.cnki.1005-4545.2020.02.35

[B55] Zhang X. Yang X. Zhang Z. Lei M. Zhang X. Wang X. (2019). Analysis of Intestinal Patients' Flora Changes with Severe Pneumonia Based on 16SrDNA Sequencing Technology. Zhonghua Wei Zhong Bing Ji Jiu Yi Xue 31 (12), 1479–1484. 10.3760/cma.j.issn.2095-4352.2019.12.009 32029033

[B56] Zhang Y. Yu W. Han D. Meng J. Wang H. Cao G. (2019). L-lysine Ameliorates Sepsis-Induced Acute Lung Injury in a Lipopolysaccharide-Induced Mouse Model. Biomed. Pharmacother. 118, 109307. 10.1016/j.biopha.2019.109307 31404772

