# Supplementary material for: Integrated Pharmacology Reveals the Molecular Mechanism of Gegen Qinlian Decoction Against Lipopolysaccharide-induced Acute Lung Injury
Source: Front Pharmacol. 2022 May 13;13:854544. doi: 10.3389/fphar.2022.854544 (PMC9136983; doi:10.3389/fphar.2022.854544)
Supplement: Supplementary file 1 [file DataSheet1.PDF]

Table 1 Primers for rat RT-qPCR

Gene	Primer sequence	GenBank accession no.	Product size (bp)
TNF-α	Forward：ATGGGCTCCCTCTCATCAGTTCC<br>Reverse：GCTCCTCCGCTTGGTGGTTTG	NM_001278601.1	228
IL-1β	Forward: CTCACAGCAGCATCTCGACAAGAG<br>Reverse：TCCACGGGCAAGACATAGGTAGC	NM_008361.4	190
IL-6	Forward：ACTTCCAGCCAGTTGCCTTCTTG<br>Reverse：TGGTCTGTTGTGGGTGGTATCCTC	NM_001314054.1	220

Table 2 Primers for rat RT-QPCR

Gene	Primer sequence	GenBank accession no.	Product size (bp)
C3	Forward：GCTGCCAACCTCATCGCCATC<br>Reverse：TGAATCACTGGTCCGTCCTCCTG	NM_016994.2	113
C5aR1	Forward：GCCCTCGCTCATTCTGCTCAAC<br>Reverse：CAGGTTACTCCGCAAGCCATCC	XM_039105863.1	147
IL17A	Forward：CTGTTGCTGCTACTGAACCTGGAG<br>Reverse：CCTCGGCGTTTGGACACACTG	NM_001106897.1	82
TGFB1	Forward: GACCGCAACAACGCAATCTATGAC<br>Reverse：CTGGCACTGCTTCCCGAATGTC	NM_021578.2	94
CYP1A1	Forward：TGGAGCCTCATGTACCTGGTAACC<br>Reverse：CGATCCCTGCCAATCACTGTGTC	XM_006243150.3	83

Table 3 NMR data attribution of metabolites in rat lung tissue

NO.	Metabolites	Assignments	Chemical shifts
1	Isoleucine	δCH_3, γCH_3, αCH	0.95(t), 1.02(d), 3.68(d)
2	Leucine	δCH_3, δCH_3, γCH, αCH	0.96(d), 0.97(d), 1.72(m)
3	Valine	γCH_3, γCH_3, βCH, αCH	1.00(d), 1.05(d), 2.26(m), 3.60(d)
4	3-Hydroxybutyrate	CH_3, CH_2	1.20(d), 2.30,2.42(dq)
5	Lactate	CH_3, CH	1.34(d), 4.12(q)
6	Alanine	βCH3, αCH	1.49(d), 3.79(q)
7	Acetate	CH_3	1.93(s)
8	Lysine	δCH_2, βCH_2, εCH_2	1.75(m), 1.92(m), 3.03(t)
9	Methionine	CH_3, CH_2	2.14(s), 2.65(t)
10	Glutamate	γCH_2, βCH_2, αCH	2.10(m), 2.36(m), 3.77(t)
11	Pyruvate	βCH_3	2.38(s)
12	Glutamine	γCH_2	2.46(m)
13	Glutathione	γCH_2	2.55(m)
14	Isocitrate	CH_2	2.53(m)
15	Aspartate	βCH_2	2.69(m), 2.82(m)
16	dimethylamine	CH_3	2.72(s)
17	Creatine	CH_3, CH_2	3.04(s), 3.94(s)
18	Ethanolamine	βCH_2	3.15(t)
19	Choline	$N(CH_3)_3$, N-CH_2, O-CH_2	3.21(s), 3.52(m), 4.07(m)
20	Betaine	$N(CH_3)_3$, CH_3	3.26(s), 3.90(s)
21	Trimethylamine N-oxide	CH_3	3.27(s)
22	Taurine	NH_2-CH_2, SO_3-CH_2	3.29(t), 3.43(t)
23	Glucose	CH, CH_2	3.38~3.56(m), 3.69~3.93(m)
24	Glycine	CH_2	3.57(s)
25	Myo-inositol	CH	3.54(dd), 4.07(t)
26	Ascorbate	CH	4.03(m), 4.52(d)
27	Uracil	CH=CH-N	5.81(d), 7.55(d)
28	Uridine	H5, H1', H_6	5.91(d), 5.93(d), 7.88(d)

29	Adenosine	H1', H2, H8	6.07(d), 8.25 (s), 8.36(s)
30	Inosine	O-CH-N, N-CH=N, N-CH=N	6.11(d), 8.25(s), 8.36(s)
31	Fumarate	CH=CH	6.53(s)
32	Tyrosine	H6, H5	6.91(d), 7.20(d)
33	Tryptophan	CH=CH	7.55(d), 7.74(d)
34	Phenylalanine	CH=CH	7.34(d), 7.39(m), 7.43(m)
35	Cytidine	H1', H5, H6	5.91(d), 6.07(d), 7.85(d)
36	Xanthine	NH=CH-N	7.96(s)
37	Carnosine	CH	8.13(s)
38	Hypoxanthine	NH=CH-N, N=CH-NH	8.21(s), 8.23(s)
39	Formate	CH	8.46(s)
40	Nicotinamide/Nicotinurate	H2/H4/H5/H6	7.61(m), 8.72(m), 8.95(s)

Abbreviations: s, singlet; d, doublet; t, triplet; dd, doublet of doublets; m, multiplet; q, quartet; dq, doublet of quartets;

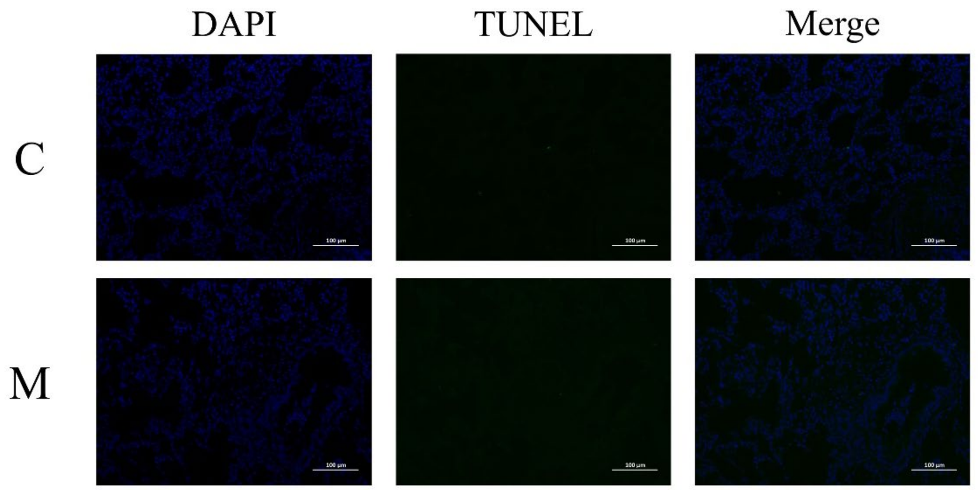

Supplement Figure 1. TUNEL detects cell apoptosis in rat lung tissue (Magnification 200×, n=6). C is a blank group. M is a model group.
